# Need assessment for the content of educational programs about breast cancer from the viewpoint of unaffected women

**DOI:** 10.1186/s12905-023-02238-x

**Published:** 2023-03-08

**Authors:** Sadaf Alipour, Marzieh Orouji, Yas Eskandari, Amirhossein Eskandari

**Affiliations:** 1 Breast Disease Research Center, Tehran University of Medical Sciences, Tehran, Iran; 2 Department of Surgery, Arash Women’s Hospital, Tehran University of Medical Sciences, Tehran, Iran; 3 Nursing Department, Arash Women’s Hospital, Tehran University of Medical Sciences, Tehran, Iran; 4 Faculty of Psychology and Education, University of Tehran, Tehran, Iran; 5 Deputy of Education, Ministry of Health and Medical Education, Tehran, Iran

**Keywords:** Breast cancer, Education, Need assessment, Questions, Community

## Abstract

**Background:**

Breast cancer is the most common cancer among females, and early diagnosis is possible in case the patients seek medical attention on time. For this to come true, they must know about the existence and risks of the disease and be aware of the appropriate attitude and actions toward prevention or early diagnosis. However, we see that women have unanswered questions about these issues. In this study, we sought to investigate healthy women’s information needs about breast cancer from their own perspective.

**Methods:**

This prospective study was carried out by using the maximum variation sampling, and theoretical saturation to reach sample saturation. Women who came to different clinics of Arash Women’s Hospital (except the Breast Clinic) during two months were entered in the study. Participants were asked to write down all the questions and subjects they would like to be explained in a breast cancer educational program. The questions were reviewed and categorized after every fifteen consecutive forms were filled until there was not even one new question. Afterwards, all the questions were reviewed and matched based on their similarity and repeated items were eliminated. Finally, questions were organized according to their common topics and the range of details they comprised.

**Results:**

Sixty patients were included in the study, and 194 questions were gathered and categorized according to common scientific terms, resulting in 63 questions in 5 categories.

**Conclusions:**

Many studies have been conducted on breast cancer education, but none have addressed healthy women's personal queries. This study outlines the questions of unaffected women about breast cancer that need to be addressed in educational programs. The results can be used for development of educational material at community level.

*Trial registration*: This study was conducted as the preliminary phase of a study approved in Tehran University of Medical Sciences (Approval Code 99-1-101-46,455) and by the Ethics Committee of the University (Ethical Code IR.TUMS.MEDICINE.REC.1399.105).

## Background

Breast cancer is the most common type of cancer across the world, and the most frequent cause of cancer death in women [1]. In Iran also, breast cancer is the more frequent female malignancy and constitutes one fourth of all cancers in the country [2, 3]. The mean age of breast cancer is lower in Iran than in western countries, and has been shown to be around 46 years of age [4]. The age-standardized incidence rate of the disease has followed an increasing pattern in recent years, a three-fold increment from 7.5–20 to 36–52 cases in 100,000 persons has been detected from 1990 to 2016, respectively [5]. Around 30% of breast cancers are detected in advanced stages [3] and it has been shown that the 5-year and 10-year survival of breast cancer is lower in Iran compared to developed countries [6, 7].

The type of treatment, the rate of tumor regression and disease remission secondary to treatments, the quality of the patient's life, and her lifespan depend on the stage in which the cancer is diagnosed [8, 9]. Therefore, early diagnosis of the disease is highly important. This is only made possible when the patient seeks medical help in the early stages, which means before the appearance of any symptom, or as early as the first manifestations [10]. On the other hand, some lesions of the breast precede the manifestation of breast cancer, and timely diagnosis of these precancerous lesions can assist in breast cancer prevention [11–14]. For this early detection to happen, it is necessary for women to be examined in appropriate times but this will only be possible if women are aware of this necessity. This is why breast cancer awareness programs are being run in many countries [15].However, data that are included in these programs might not be enough, and interestingly, health messages may be interpreted differently in various cultures [16].

Some studies in Iran have investigated the levels of breast cancer awareness and the source of women’s information about this disease. As expected, there were several deficiencies in women’s education, as the social, religious and political circumstances of the country do not allow for openness when talking about diseases of the breast, especially in large scales or in mass media. In a large population-based study held by Montazeri et al. [17] in Iran to assess the knowledge about breast cancer among 1402 women (mean age 43.4 ± 14.4 years) without a history of breast cancer and with no relative affected with the cancer, around 60% of participants had heard about breast cancer and screening, and about 30% and 15% had heard about it on TV or radio programs. However, the knowledge about presenting signs and screening methods was highly inadequate in the majority of participating women. A systematic review [18] of the existing literature up to September 2017, investigating the knowledge of women in Iran regarding the early diagnosis of breast cancer and their sources of information, found 25 eligible studies collectively including more than 11,700 people. Overall, this study detected a low level of knowledge derived from various sources, the most common source being healthcare staff. More recent studies that assessed the obstacles against opportunistic breast cancer screening in Iran showed that lack of awareness, as well as embarrassment and religious beliefs were the more common barriers [19, 20]. Interestingly, one of the important sources of information and awareness propagation for breast cancer in Iran is through the activities of Cancer Charities [21, 22]; although these activities need to be organized in order to affect the awareness of a large portion of the population [22].

Women that are aware of the risk of breast cancer may have unanswered questions and concerns about various aspects of the disease. This state of confusion can cause unreasonable worry and fear of the disease and leads to not taking action at the proper time [17, 23, 24]. Answering these questions alongside spreading appropriate breast cancer-related education can help erase some of the irrational worries and fears.

Generally, women who are undergoing breast cancer treatment, as well as breast cancer survivors, are expected to be aware of the management options and the course and prognosis of the disease [25]. Also, women harboring benign breast diseases that might increase the risk of breast cancer are likely to be aware of this increased risk and should know about the probable methods to decrease this risk or increase the chance of early diagnosis [26].Normally, all this information is expected to be provided by the caring team for patients with malignant or benign breast diseases; though this does not happen regularly[26, 27]. However, women with healthy breasts, who constitute the largest proportion of the female population and are the target group of screening programs for early diagnosis of high-risk or cancerous lesions, might not be exposed to data regarding breast cancer and early diagnosis; unless it is provided through educational programs. Furthermore, those who have heard about breast cancer or received data about it might have questions that have not been answered through those instructions. These questions should be sought through a methodical approach in order to provide an accurate account of the information needs of these women.

Considering that many health centers and scientific or social institutions and charities have an inclination to support women’s wellbeing by offering different forms of educational material about health topics, we decided to carry out a precise and thorough study to find all the frequent, and also less common questions of healthy women about breast cancer. Thus, the main items that should constitute the basic template of educational packages aiming to satisfy information concerns about breast cancer would be defined.

## Methods

This study was conducted as the preliminary phase of a study approved in Tehran University of Medical Sciences (Approval Code 99-1-101-46,455) and by the Ethics Committee of the University (Ethical Code IR.TUMS.MEDICINE.REC.1399.105) [28]. Informed consent was obtained from all subjects. The work was performed in accordance with the ethical standards of the ethical committee of the University on human experimentation and with the Helsinki Declaration (as revised in 1983).

This prospective study was carried out according to the grounded theory method, by using the maximum variation sampling, and theoretical saturation to reach sample saturation. Women who came to different clinics of Arash Women’s Hospital except the Breast Clinic between 10 to 12 o'clock in the morning (the busy hours of the clinics) during two months were entered in the study.

After being given a thorough explanation about the subject and aim of the study by two of the clinic nurses and obtaining informed consent, the women were asked to write down all of their questions regarding breast cancer in pre-prepared forms. They were told to write what questions they needed an answer to if an educational program dedicated to breast cancer was designed for them.

In the cases that the participants were unable to convey their concept in written form, this task was done with the help of the interviewing nurse, while other women did it by themselves. A team consisting of a breast surgeon and two breast care nurses were in charge of developing a list that would contain all the questions mentioned by participants. The questions written in the forms were reviewed by members of the team after every fifteen consecutive filled forms. The team categorized the questions according to their main theme, detected questions with similar content and meaning, deleted analogous questions, and added newly mentioned questions to the list. The process continued as long as there was a new question, and came to an end when there was not even one new question in a set of 15 successive forms. Thereafter, all the questions were reviewed and matched based on their similarity and according to their synonym scientific terms; and similar or repeated items were eliminated. Questions encompassing a wide range of subjects were categorized as general questions, and those asking about details were grouped as detailed questions. Also, questions were organized according to their common topics.

## Results

The mean age of the interviewed women was 45.5 (range 18–64) years. Around half of the participants were above 40 years and about 20% were above 50; these were in the mammographic screening age based on different guidelines [29, 30]. After four sequences of collecting samples, no new questions were posed in the 15 questionnaires of the last round. The number of the women in the study totaled 60 people. The number of questions in each form was from 1 to 14 questions, with the mean of 3 questions; and a total of 194 questions. After organizing the questions depending on whether they were general or detailed, and categorizing them based on shared topics, a total of 63 questions in 5 categories were reached, which are shown in Table 1.

**Table 1:** Questions of healthy women about breast cancer based on generality and subject

Number	Group	Question
*General items*		
1	General	What are the stages of breast cancer ?
2	General	Do men get involved with breast cancer?
*Causes (risk factors)*		
3	General	Why does breast cancer happen ?
4	General	What are the risk factors for breast cancer ?
5	Detailed	Is breast cancer related to pregnancy and breastfeeding ?
6	Detailed	Is menopause related to breast cancer ?
7	Detailed	Are breast cysts related to breast cancer ?
8	Detailed	Does stress induce or increase breast cancer?
9	Detailed	Does removal of unwanted hair by laser increase breast cancer?
10	Detailed	Is marriage related to the risk of breast cancer?
11	Detailed	Do breast implants increase breast cancer?
12	Detailed	Do contraceptive pills increase breast cancer?
13	Detailed	Do hormonal medicine increase breast cancer?
14	Detailed	Do chemical compounds increase breast cancer?
15	Detailed	Do herbal medicines affect the risk of breast cancer?
16	Detailed	Does lifestyle affect the risk of breast cancer?
17	Detailed	Is nutrition related to breast cancer?
18	Detailed	Doe sports affect the risk of breast cancer?
*Manifestations*		
19	General	How does breast cancer present?
20	Detailed	Are all breast masses cancerous?
21	Detailed	Is the alternating inward position of the nipple dangerous?
22	Detailed	Is breast itching a sign of breast cancer ?
23	Detailed	Is pus discharge from the nipple a sign of breast cancer ?
24	Detailed	Are lumps in the armpit a sign of breast cancer ?
25	Detailed	Is a larger breast related with breast cancer ?
26	Detailed	Is breast pain a sign of breast cancer ?
27	Detailed	Is bleeding from the nipple a sign of breast cancer ?
28	Detailed	Is water discharge from the nipple a sign of breast cancer ?
*Diagnosis*		
29	General	How is breast cancer identified?
30	General	What I can do to avert breast cancer?
31	General	Do I have to exam my breasts by myself?
32	General	What should I care for when I examine my breasts by myself?
33	Detailed	When should I examine my breasts?
34	Detailed	How should I examine my breasts ?
35	General	How frequently shoud I examine my breasts?
36	General	What is the best imaging option for detection of breast cancer?
37	General	What is mammography and how is it performed?
38	General	Is undergoing mammography dangerous for me?
39	General	What are the advantages of mammography?
40	Detailed	Is ultrasound of the breast dangerous for the baby if I am pregnant?
41	General	Can MRI replace mammography for finding breast cancer?
42	General	Why breast sampling is performed?
43	General	How is needle breast sampling performed?
44	Detailed	Is needle breast sampling performed under local or general anesthesia?
45	Detailed	If the doctor orders breast sampling, does it mean that I have cancer?
46	Detailed	Why surgeons perform some samplings by themselves, but refer others to radiologists?
47	Detailed	Why the doctor orders breast sampling while the imaging has shown benign disease?
48	Detailed	Does breast needle sampling cause enlargement of the breast cancer?
49	Detailed	Does needle sampling changes a benign lump to a malignant one?
50	Detailed	Does breast needle sampling cause cancer dissemination?
*Treatment*		
51	Detailed	Is complete removal of the breast needed for breast cancer cure?
52	Detailed	Is breast reconstruction possible at the time of removing the breast?
53	Detailed	Does the breast get distorted and ugly after breast surgery for cancer?
54	Detailed	Does the arm always swell after breast cancer surgery, and is it lifelong?
55	Detailed	Is strict restriction of arm movements needed after breast cancer surgery?
56	General	Do all breast cancers need chemotherapy?
57	Detailed	Does chemotherapy always cause baldness?
58	General	Do all breast cancers need radiation?
59	General	Is chemotherapy better for breast cancer treatment or radiation?
60	General	What is hormone therapy for breast cancer?
61	General	What should the patient do after breast cancer treatment?
62	General	How could breast cancer be stopped?
63	General	What is the benefit of early diagnosis in breast cancer treatment?

## Discussion

In this study, the questions of healthy women about breast cancer were collected and classified with the intent to be used in designing focused educational material.

Many studies have investigated the level of awareness of the general population about breast cancer, and its relation with undergoing breast cancer screening; highlighting that breast cancer awareness is one of the concerns of researchers in most countries [15, 31–33]. These and other studies confirm that despite some educational programs being carried out, there is still a significant lack of information about breast cancer among women [15, 17, 34]. However, few studies have focused on the topics that healthy women are unfamiliar with, and the questions the individuals themselves have; the investigation of the informational needs was a secondary goal in those works [17, 35–37]. In a study carried out by Montazeri et al. [17] on 1402 women, it was revealed that lack of information about breast cancer was most prominent when it came to the two major topics of disease symptoms (painless lumps, retraction, and bloody nipple discharge) and the effective methods of screening. A study on middle school teachers by Kalan et al. [35] measured the effect of breast-cancer-related education on the awareness level and attitude of the participants. The authors’ conclusive advice was for programs with the aim of educating women about timely diagnosis. Grunfeld et al. [36] evaluated women’s knowledge about breast cancer. The results showed that awareness level about risk factors, the risk of the disease, the rate of personal risk and the number of symptoms was low, especially in older women. In a systematic review by Gupta et al. [37], the awareness levels of Indian women and health system staff about the risk factors of breast cancer were studied. In total the information of more than 700 people was collected. The results showed that information regarding breast cancer’s risk factors is lacking in the general population.

Numerous researches have been performed about knowledge gaps and information needs in breast cancer patients [25, 38–41]. Nevertheless, this matter has not been studied in depth in healthy women. Our study is the first to approach the issue from the point of view of unaffected women, to see their knowledge deficits from their perspectives, and to explore their own queries. The importance of understanding the information needs of healthy women is that they are the main target group who will benefit from screening, as women already affected by breast cancer would not take great advantage from being educated about breast cancer early diagnosis. This group learn some points from their own disease and the management they have received, while also being instructed about breast cancer by their health care providers and having access to living resources of information about the disease. Though they still need to be educated more efficiently, and studies have investigated their information needs and sources regarding their disease status, management options, prognosis, and quality of life as a survivor [27, 41–43]. However, healthy women are only targeted through specific educational programs or by information distributed in mass media. These programs might not contain the answer to their questions, because to our knowledge, no study has investigated this issue up to now. On the other side, in patients affected by known high-risk benign breast diseases, the approach and management offered by the health-care provider gives some significant information to the patients [14, 26]. A literature search, however, shows no specific study aiming at the knowledge gaps in this group. Their questions might be similar to those of healthy women as detected in our study, although this should be assessed independently; the method and results of our study would serve as a basis for such a research.

Interestingly, in a systematic review by Austoker et al. [15], the effect of breast-cancer-related education on the person’s awareness and their subsequent behavior to take early action was not shown to be directly related. We believe that paying attention to the women’s own questions and the subjects they themselves want to know about could increase the efficacy of such education and have a positive effect on the patients’ proactive behavior as well as reduce their delay in taking action.

In brief, none of the present studies have addressed the informational needs of women from their own point of view or studied the women’s questions about breast cancer with the aim of answering their own questions in subsequent educational programs. Our study has addressed this issue and sought the women’s concerns about breast cancer, and the questions they want to be answered. The findings of this study can be used in any local or community-based education program for raising women’s awareness and calming their worries about breast cancer.

### Limitations

This study had few limitations. We did not gather data about the level of education, the socioeconomic status, and the occupation of the participants. Also, it was held in a single center. Thus, we suggest that this need assessment be carried out via similar studies by including a wide range of participants regarding education and socioeconomic levels in a multi-centric setting, and also including women with benign breast diseases.

## Conclusions

This study defines and categorizes the frequent and infrequent questions of healthy women about breast cancer. These can be used for designing effective educational material in local, nationwide, and international programs. Leading similar studies in a wider range and in multi-centric designs is suggested.

## Data Availability

The datasets used during the current study is available from the corresponding author on reasonable request.
